# Supplementary material for: TET-mediated epimutagenesis of the Arabidopsis thaliana methylome
Source: Nat Commun. 2018 Mar 1;9:895. doi: 10.1038/s41467-018-03289-7 (PMC5832761; doi:10.1038/s41467-018-03289-7)
Supplement: Supplementary file 1 — Supplementary Information [file 41467_2018_3289_MOESM1_ESM.pdf]

Table S1. Methylome sequencing summary statistics

Sample	Length (bp)	Mapped reads	Non-conversion (%)	Genome coverage	Coverage per-strand
Col-0 WT-1	150	25,858,836	0.30%	32.5	16.3
Col-0 WT-2	150	20,708,437	0.17%	26.0	13.0
35S:TET1-1	150	11,483,070	0.17%	14.4	7.2
35S:TET1-2	150	25,300,675	0.12%	31.8	15.9
35S:TET1-3 rep1	150	15,239,451	0.16%	19.2	9.6
35S:TET1-3 rep2	150	13,976,359	0.13%	17.6	8.8
35S:TET1-2 ^{T5}	150	10,149,189	0.19%	12.8	6.4
35S:TET1-2 ^{T6}	75	46,968,226	0.12%	29.5	14.8
35S:TET1-1.1 $^{+TET1}$	150	21,161,361	0.11%	26.6	13.3
35S:TET1-1.2 $^{+TET1}$	150	24,145,320	0.09%	30.4	15.2
35S:TET1-1.3 $^{-TET1}$	150	24,782,835	0.11%	31.2	15.6
35S:TET1-1.4 $^{-TET1}$	150	21,725,685	0.30%	27.3	13.7
35S:TET1-1.5 $^{-TET1}$	150	23,032,079	0.25%	29.0	14.5
ACT2:TET1-1.1 $^{+TET1}$	150	25,564,705	0.16%	32.1	16.1
ACT2:TET1-1.2 $^{-TET1}$	150	24,743,874	0.15%	31.1	15.6
ACT2:TET1-2	75	35,548,625	0.12%	22.3	11.2
ACT2:TET1-3	75	34,292,795	0.12%	21.6	10.8
ACT2:TET1-4	75	42,867,331	0.16%	26.9	13.5
ACT2:TET1-5	75	36,877,672	0.47%	23.2	11.6

Table S2. Transriptome sequencing summary statistics

Sample	Mapped reads	Percent Mapped
Col-0 WT rep1	18,065,032	95.39%
Col-0 WT rep2	24,156,251	96.63%
Col-0 WT rep3	20,987,335	96.48%
35S:TET1-1	19,919,035	95.38%
35S:TET1-2	15,977,645	96.51%
met1	45,615,533	94.61%
ibm1 rep1	20,810,937	94.16%
ibm1 rep2	22,011,997	96.17%
ibm1 rep3	25,241,389	97.05%

Table S3. TAB-seq summary statistics

	Mapped reads	5mC non-conversion (%)	Non-conversion (%)	Genome coverage
Col-0 WT	15,044,614	3.97%	0.28%	18.9
35S:TET1-3 rep1	14,293,516	4.69%	0.28%	18.0
35S:TET1-3 rep2	16,145,140	4.68%	0.29%	20.3

Table S4. ChIP-seq summary statistics

	Uniquely mapped reads	Non-clonal reads
35S:TET1-2 ^{T6}	6,754,041	4,639,956

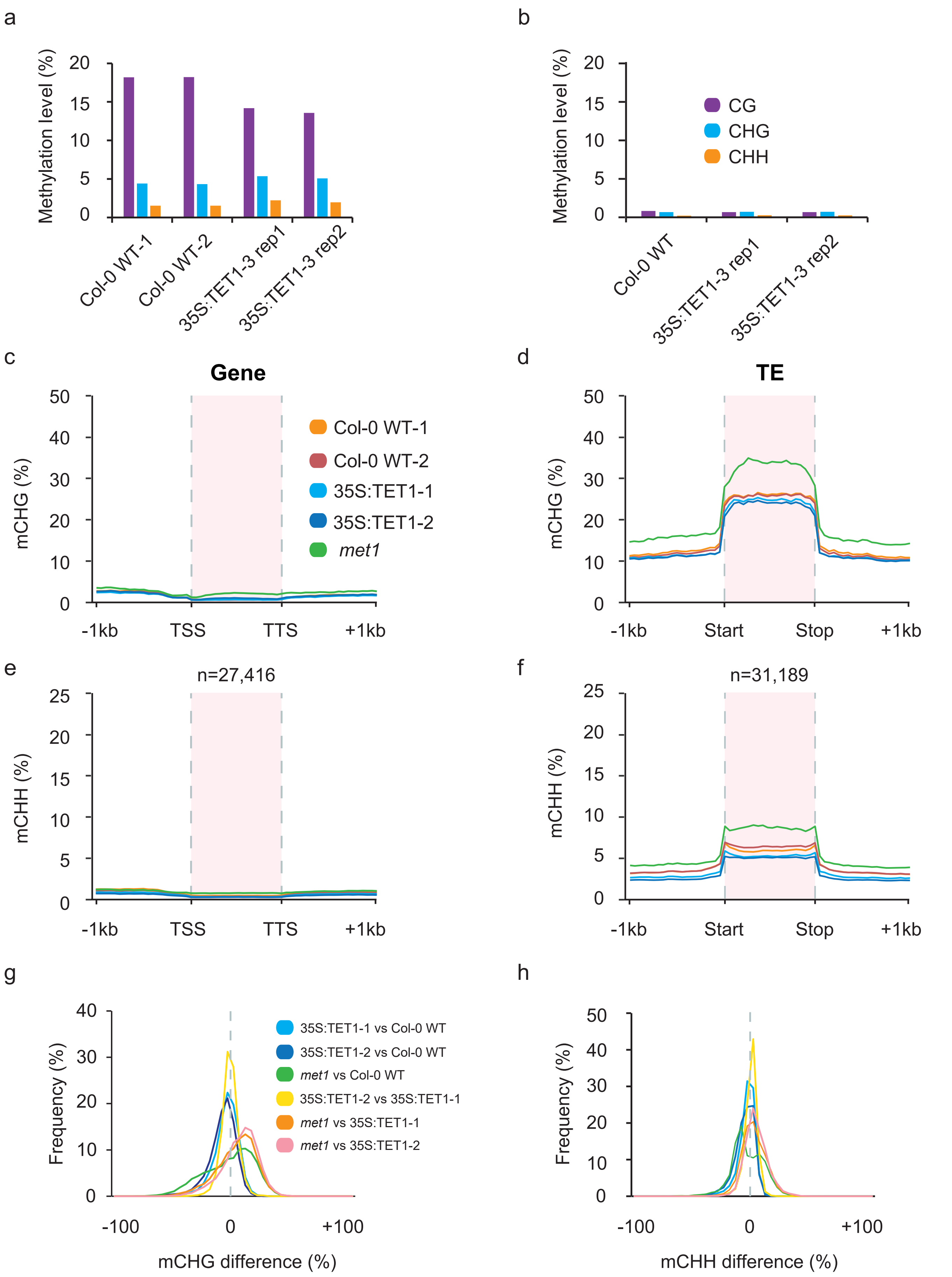

Figure S1. Global methylation (a) and 5hmC (b) levels of Col-0 WT plants and 35S:TET1 transgenic plants. Metagene plots of CHG (c and d) and CHH (e and f) methylation level across gene bodies and transposable elements. Frequency plots of mCHG (g) and mCHH (h) differences in CG DMRs that posses non-CG methylation in wild type. The average value of two Col-0 WT plants was defined as the Col-0 WT.

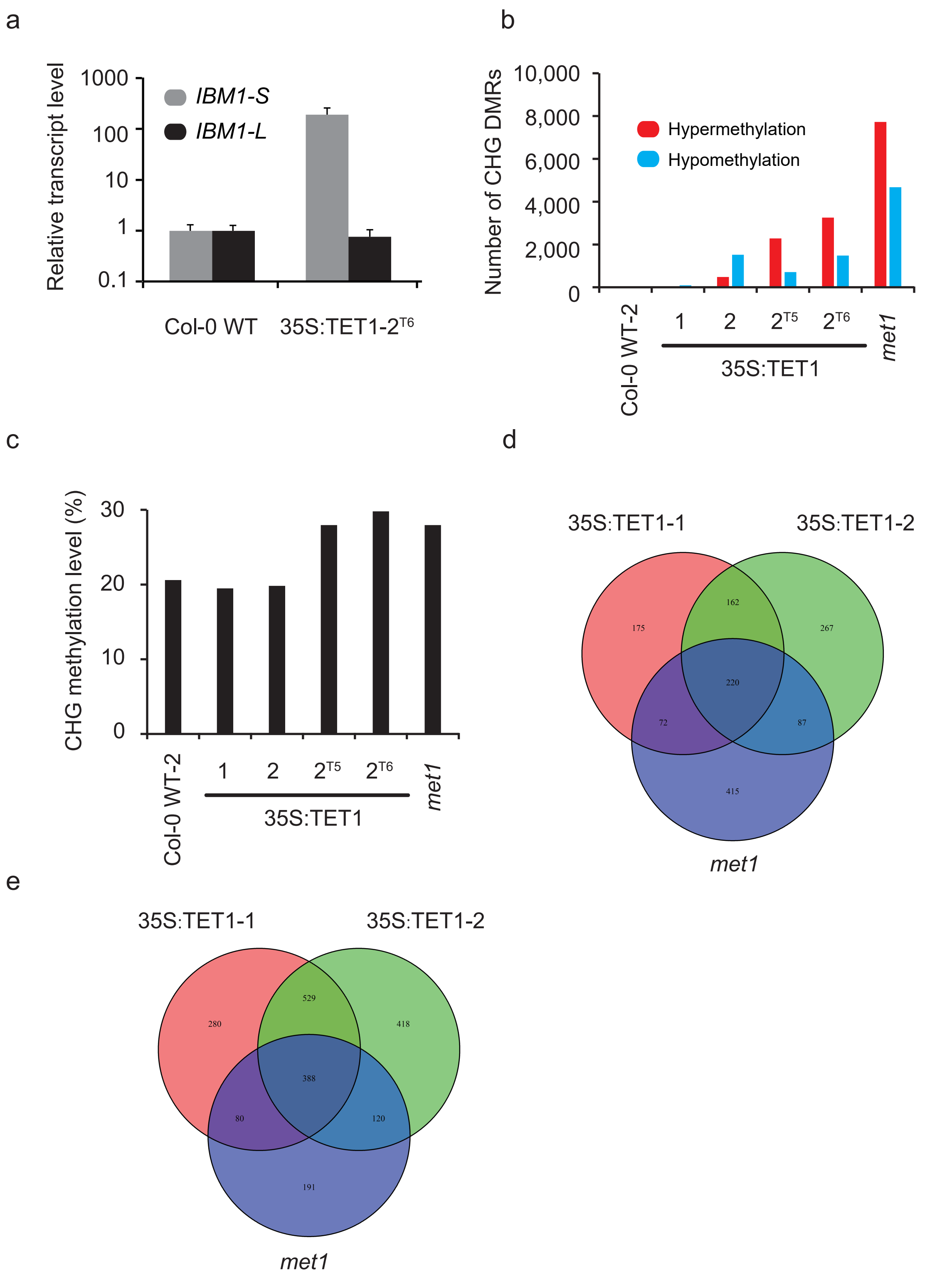

Figure S2. (a) Relative mRNA expression of *IBM1-L* and *IBM1-S* in Col-0 WT and 35S:TET1-2^{T6} examined by RT-qPCR. Error bars indicate s.d. (b) Number of identified CHG DMRs in 35S:TET1 individuals. These DMRs were defined relative to Col-0 WT-1, as Col-0 WT-2 DMRs were used to assess background interference. (c) Bar plot of CHG methylation levels in identified CHG DMRs. Venn diagrams of upregulated genes (d) and downregulated genes (e) in two 35S:TET1 transgenic individuals and *met1*.

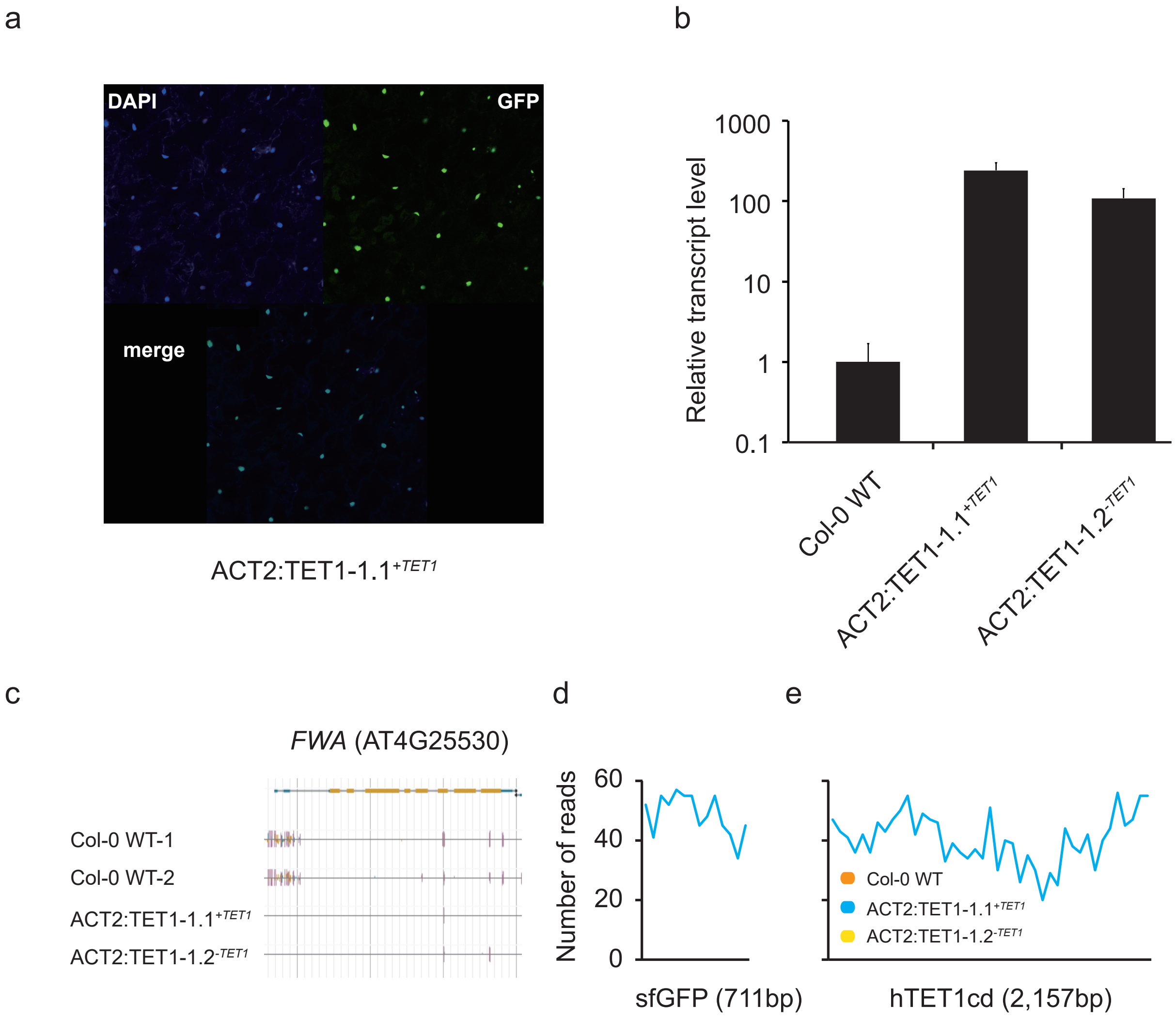

Figure S3. (a) Confocal microscopy of sfGFP-TET1cd fusion protein in young cotyledons. DAPI and GFP stains show the locations of nuclei and GFP signal, respectively. (b) Relative expression of FWA examined by RT-qPCR. Error bars indicate s.d. (c) Genome browser view of *FWA* (AT4G25530). Read distribution (50bp windows) in (d) sfGFP and (e) hTET1cd sequences indicate the absence of sfGFP-hTET1cd in Col-0 WT and ACT2:TET1-1.2^{-TET1}.

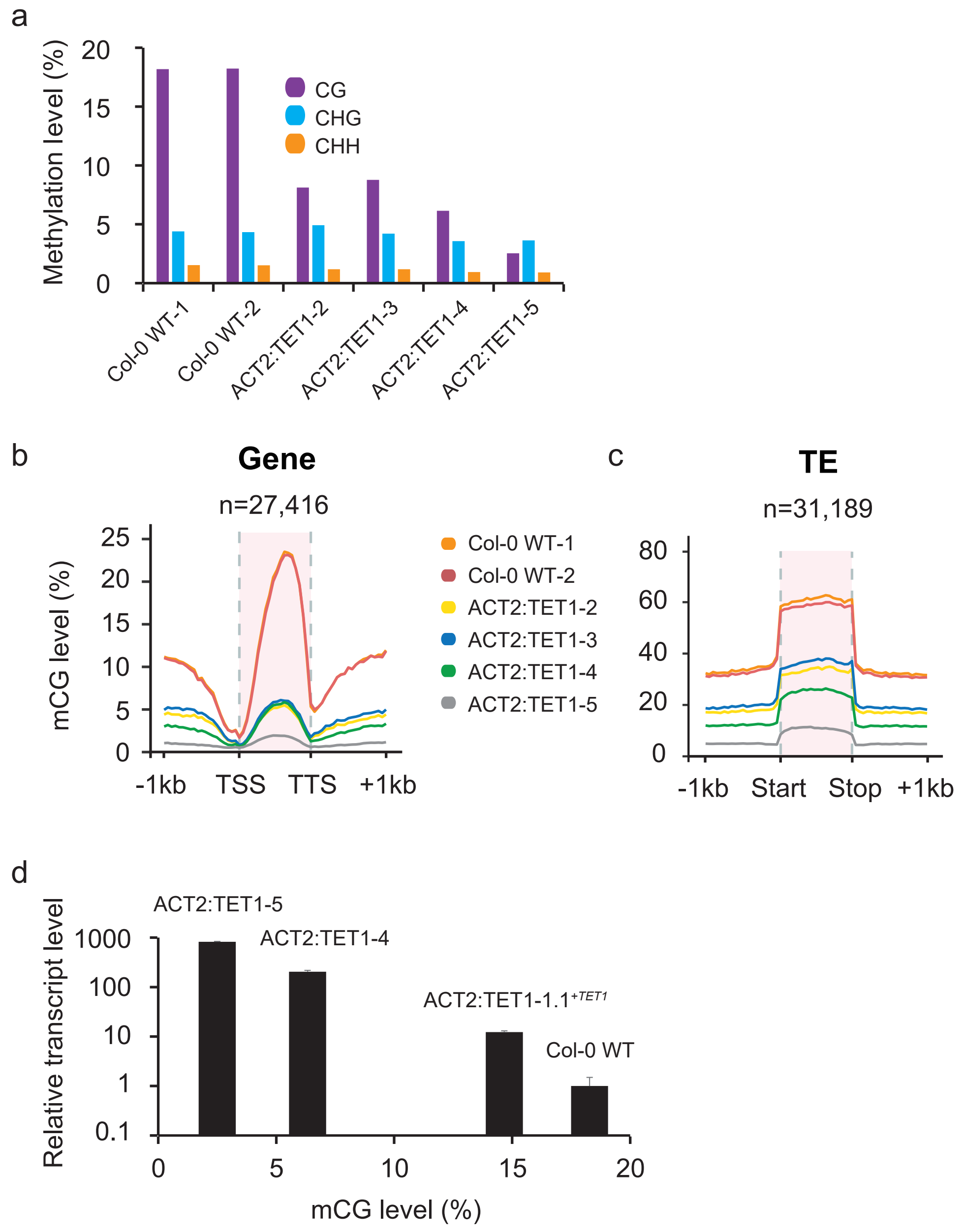

Figure S4. (a) Bar plot of global methylation levels in two Col-0 WT replicates and four ACT2:TET1 T1 plants. Metagene plots of CG methylation level across (b) gene bodies and (c) transposable elements. (d) Relative expression of sfGFP-hTET1cd examined by RT-qPCR and corresponding mCG level indicate a relationship between transgene expression level and mCG loss. Error bars indicate s.d.
