# Supplementary material for: TET-mediated epimutagenesis of the Arabidopsis thaliana methylome
Source: Nat Commun. 2018 Mar 1;9:895. doi: 10.1038/s41467-018-03289-7 (PMC5832761; doi:10.1038/s41467-018-03289-7)
Supplement: Supplementary file 2 — Description of Additional Supplementary Files [file 41467_2018_3289_MOESM2_ESM.pdf]

Description of Supplementary Files

File Name: Supplementary Data 1
Description: **CG methylation levels of transposons in wild type and 35S:TET1 lines**. CG methylation levels were calculated for all annotated transposons in wild type and in 35S:TET1 lines.

File Name: Supplementary Data 2
Description: **CG methylation levels of genes in wild type and 35S:TET1 lines**. CG methylation levels were calculated for all annotated genes in wild type and in 35S:TET1 lines.

File Name: Supplementary Data 3
Description: **TET1 induced DMRs**. Methylation levels (CG, CHG and CHH) for differentially methylated regions (DMRs) identified between wild type and 35S:TET1 lines.

File Name: Supplementary Data 4
Description: **ACT2:TET1 CG DMRs**. CG methylation levels in CG DMRs identified between four ACT2:TET1 T1 lines.

File Name: Supplementary Data 5
Description: **ACT2:TET1 CHG DMRs**. CHG methylation levels in CHG DMRs identified between four ACT2:TET1 T1 lines.

File Name: Supplementary Data 6
Description: **ACT2:TET1 CHH DMRs**. CHH methylation levels in CHH DMRs identified between four ACT2:TET1 T1 lines.
